# How to Measure and Calculate Equivalent Series Resistance of Electric Double-Layer Capacitors

**DOI:** 10.3390/molecules24081452

**Published:** 2019-04-12

**Authors:** Rafael Vicentini, Leonardo Morais Da Silva, Edson Pedro Cecilio Junior, Thayane Almeida Alves, Willian Gonçalves Nunes, Hudson Zanin

**Affiliations:** 1 Advanced Energy Storage Division, Center for Innovation on New Energies, Carbon Sci-Tech Labs, School of Electrical and Computer Engineering, University of Campinas, Av Albert Einstein 400, Campinas, SP 13083-852, Brazil; rafael.vicentini22@gmail.com (R.V.); edbig@uol.com.br (E.P.C.J.); thayaneaalves@gmail.com (T.A.A.); nuneswillian40@gmail.com (W.G.N.); 2 Department of Chemistry, Federal University of Jequitinhonha e Mucuri’s Valley, Highway MGT 367, km 583, 5000, Alto da Jacuba, Diamantina, MG 39100-000, Brazil

**Keywords:** equivalent series resistance, galvanostatic charge-discharge method, impedance technique, voltage drop, simulation of equivalent circuit models

## Abstract

Electric double-layer capacitors (EDLCs) are energy storage devices that have attracted attention from the scientific community due to their high specific power storage capabilities. The standard method for determining the maximum power (*P*_max_) of these devices uses the relation *P*_max_ = *U*^2^/4*R*_ESR_, where *U* stands for the cell voltage and *R*_ESR_ for the equivalent series resistance. Despite the relevance of *R*_ESR_, one can observe a lack of consensus in the literature regarding the determination of this parameter from the galvanostatic charge-discharge findings. In addition, a literature survey revealed that roughly half of the scientific papers have calculated the *R*_ESR_ values using the electrochemical impedance spectroscopy (EIS) technique, while the other half used the galvanostatic charge discharge (GCD) method. *R*_ESR_ values extracted from EIS at high frequencies (>10 kHz) do not depend on the particular equivalent circuit model. However, the conventional GCD method better resembles the real situation of the device operation, and thus its use is of paramount importance for practical purposes. In the latter case, the voltage drop (Δ*U*) verified at the charge-discharge transition for a given applied current (*I*) is used in conjunction with Ohm’s law to obtain the *R*_ESR_ (e.g., *R*_ESR_ = Δ*U*/Δ*I*). However, several papers have caused a great confusion in the literature considering only applied current (*I*). In order to shed light on this important subject, we report in this work a rational analysis regarding the GCD method in order to prove that to obtain reliable *R*_ESR_ values the voltage drop must be normalized by a factor of two (e.g., *R*_ESR_ = Δ*U*/2*I*).

## 1. Introduction 

From urban mobility to portable electronics, fast charging devices are in growing demand [1,2,3]. In this scenario, electric double-layer capacitors (EDLCs), also known as supercapacitors, are very attractive energy storage devices with ultra-fast and short-term features [2,3,4]. To scale the characteristics of different EDLCs, the specific energy and power are commonly evaluated for practical purposes. The key parameters for the energy storage devices are regarded to the energy (Wh kg^−1^) and power (W kg^−1^) normalized per weight of the device (or the electrode material). In this sense, the maximum output energy (*E*_max_) and power (*P*_max_) are determined using the relations *E*_max_ = *CU*^2^/2 and *P*_max_ = *U*^2^/4*R*_ESR_, respectively, where *U* stands for cell voltage, *C* for specific capacitance, and *R*_ESR_ for equivalent series resistance (ESR) [5].

It is commonly verified in the literature that ESR can be properly determined using the electrochemical impedance spectroscopy (EIS) and the galvanostatic charge-discharge methods. In the case of EIS, the ESR value can be accurately determined at high frequencies (>10 kHz) [6,7,8]. This method is quite simple to be applied since it does not rely on the use of a particular equivalent circuit model for the device [9]. Despite the advantages offered by the EIS method, we must consider that the conventional GCD method better resembles the real operation conditions for supercapacitors, i.e., the use of this method in the laboratory studies is of paramount importance in order to obtain a more realistic experimental scenario resembling a real application.

In the case of the GCD method, the voltage drop (Δ*U*) verified at the charge-discharge transition for a given applied current (*I*) must be used in conjunction with Ohm’s law to obtain the *R*_ESR_ (e.g., *R*_ESR_ = Δ*U*/Δ*I* and not *R*_ESR_ = Δ*U*/*I*) [5,10,11,12,13,14,15,16]. However, the ‘ad hoc’ adoption in the literature of non-standard normalizing factors for Δ*U* have led to a great confusion when findings present in different papers obtained for supercapacitor devices were compared.

The objective of the present work is to present a theoretical analysis of the GCD method to demonstrate that correct determination of *R*_ESR_ values requires the voltage drop to be normalized by a factor of two (e.g., *R*_ESR_ = Δ*U*/2*I* and not *R*_ESR_ = Δ*U*/*I*). Simulations using canonic circuit models were carried out to emphasize the theoretical aspects inherent to the present work. Furthermore, a comparison of the theoretical electrochemical behaviors of these circuits in the frequency domain is presented using the EIS method.

## 2. Fundamentals of the EIS and GCD Methods

**Figure 1:**
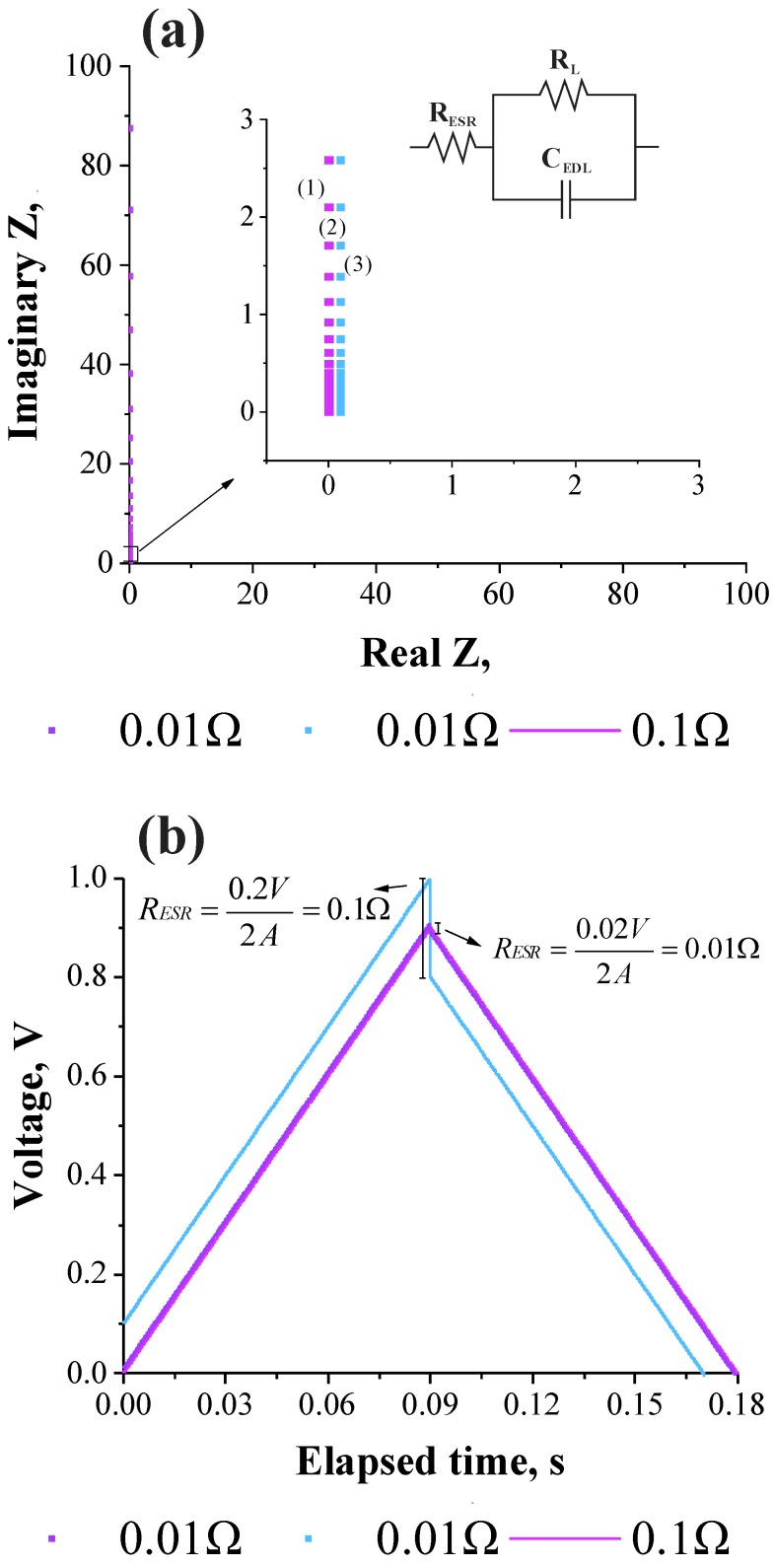
Schematic representation of (**a**) the complex-plane plots and (**b**) the galvanostatic charge-discharge curves evidencing the voltage drop (*U*_drop_). The inset in Figure 1a shows the canonic circuit model. Simulation was carried out considering different values of the *R*_ESR_ and *R*_L_ = 1 MΩ.

Figure 1a,b shows the simulated Nyquist (e.g., complex-plane) plots and the corresponding galvanostatic charge-discharge plots obtained as a function of the *R*_ESR_. It is worth mentioning that the impedance response verified at very high frequencies does not depend on the particular equivalent circuit model used in the simulation process [7,8,17,18,19,20,21,22]. In addition, for practical purposes, since the EIS is a steady-state technique obeying the linear theory of systems, one has that highly accurate values of resistances and capacitances can be obtained for EDLCs using a low amplitude sinusoidal voltage (e.g., δ*U* = 10 mV (peak-to-peak)) and scanning the frequency for various orders of magnitude (e.g., Δ*f* = 100 kHz to 10 mHz).

From the above considerations, simulations were accomplished using a canonic equivalent circuit model representing the ideal electrochemical response of a symmetric coin cell device where the positive and negative electrodes are identical. As it can be seen in the inset of Figure 1, the equivalent series resistance (*R*_ESR_) is connected to a branch containing a capacitor (*C*_EDL_), representing the charge storage process on the electrical double-layer (EDL) formed at the electrode/electrolyte interface, which stands in parallel with a leakage resistance (*R*_L_). Despite the use of a canonic model, it is important noting that the *R*_ESR_-value obtained at high-frequencies always appear as a generalized resistance connected in series with the other circuit elements of the particular circuit model [4].

The simulation presented in Figure 1a was accomplished for different *R*_ESR_-values and using an *R*_L_ (impedance to leak current)-value of 1 MΩ. Obviously, an ideal EDLC device has a very high leakage resistance (*R*_L_ → ∞) and the phenomenon of frequency dispersion is absent, i.e., the complex-plane plot is characterized by a perfect vertical line (see Figure 1a). Thus, the extrapolation of this line on the real axis (*Z*_real_) at very high frequencies (e.g., ω → ∞) yields the desired value of the *R*_ESR_. Therefore, in real systems the true value of *R*_ESR_ is commonly determined by extrapolation using a high-frequency value of ≈1.0 kHz.

Figure 1b shows the galvanostatic charge-discharge curves evidencing the voltage drop (*U*_drop_) at the inversion of polarity. From the theoretical viewpoint, in this case the determination of the *R*_ESR_-value involves the application of a square wave current function with the inversion in polarity (e.g., *I*_(+)_ ↔ *I*_(−)_ and |*I*_(+)_| = |*I*_(−)_|).A voltage drop is observed at the reversal of polarization with a voltage increase after the sign of the current was reversed. During a continuous repetition of the charge–discharge processes, the positive (anode) and negative (cathode) electrodes were constantly charged and discharged, respectively, for equal times by applying positive (*I*_(+)_) and negative (*I*_(−)_) currents of the same magnitude (|*I*_(+)_| = |*I*_(−)_|). Therefore, for an ideal case where only a capacitive behavior exists (*R*_ESR_ = 0) one would obtain as the response a symmetric triangular voltage wave since the capacitive voltage (*U*_c_) increases linearly with the stored charge (*Q*) for a given capacitance (*C*), i.e., δ*U*_c_ = δ*Q*/*C*. However, in real cases where *R*_ESR_ > 0 the anodic branch (e.g., the straight line with a positive slope) of the voltage wave referring to the charging process (e.g., δ*U*_c(+)_ = δ*Q*_(+)_*/C*) is displaced to more positive values by a constant value dictated by *U*_ESR(+)_ = *R*_ESR_ × *I*_(+)_ = constant. Therefore, the instantaneous values of the overall voltage are given by *U*_i_ = *U*_c(+)_ + *U*_ESR(+)_. Accordingly, the cathodic branch (e.g., the straight line with a negative slope) of the voltage wave associated with the discharging process (e.g., δ*U*_c(−)_ = δ*Q*_(−)_/*C*) is displaced to more negative voltages due to the reversal in polarity of the applied current where δ*I* = *I*_(+)_ − *I*_(−)_ = 2*I*, since |*I*_(+)_| = |*I*_(−)_|.

Intuitively, the instantaneous values of the overall voltage after the reversal in polarity is given by *U*_i_ = *U*_c_ − *U*_ESR_ = *U*_c_ − *R*_ESR_ × 2*I*. In this sense, the overall voltage drop during reversal of the polarization is *U*_ESR_ = − *R*_ESR_ × 2*I* since Δ*U*_ESR_ < 0, i.e., *R*_ESR_ = Δ*U*_ESR_/2*I* and, therefore, the voltage drop must be normalized by a factor of 2 [5].

## 3. Theoretical Electric Response of the GCD Curves Using the Canonic Equivalent Circuit Model

### 3.1. Deriving the Theoretical Formula for the Equivalent Series Resistance

**Figure 2:**
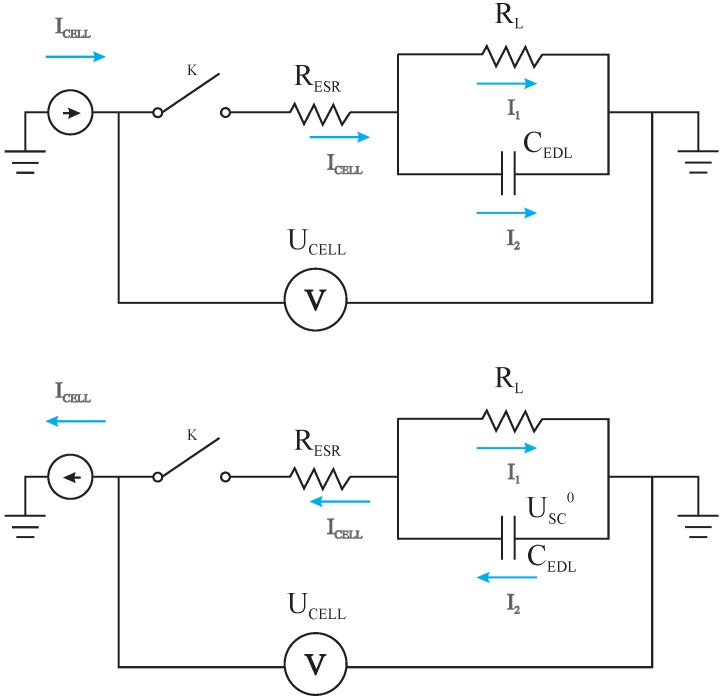
Electric circuits containing the canonic equivalent circuit model representing the electrochemical behavior of electric double layer capacitors during the charging and discharging processes carried out at constant current. Definitions: *I*_cell_ is the constant current applied; *R*_ESR_ is the equivalent series resistance; *R*_L_ is the leakage resistance; *C*_EDL_ represents the equivalent capacitance of the symmetric coin cell; *U*_cell_ is the overall cell voltage and *U*_SC_^0^ is the voltage across the *C*_EDL_ when the capacitor is fully charged.

To obtain the theoretical model for the calculation of the *R*_ESR_, the canonic circuit presented in Figure 2 representing the charge–discharge processes was employed to obtain the pertinent equations. In short, key equations for the charge and discharge processes and a combination of them are presented to obtain the desired theoretical model.

According to the basic laws governing the theory of electric circuits the cell voltage during a charging process for the circuit presented in Figure 2 is given by the following relationship:

(1) $$U_{\mathrm{cell}(t)}=R_{\mathrm{ESR}}I_{\mathrm{cell}}+R_{L}I_{\mathrm{cell}}\times \left[1-\exp\left(\frac{-t}{R_{L}C}\right)\right]$$

In addition, it is possible to demonstrate that the function *U*_cell(*t*)_ describing the transient response after the switcher *K* is closed is given by the following equation:

(2) $$U_{\mathrm{cell}(t)}=U_{\mathrm{sc}}^{0}-R_{\mathrm{ESR}}I_{\mathrm{cell}}-(U_{\mathrm{sc}}^{0}+R_{L}I_{\mathrm{cell}})\times \left[-\;1+\exp\left(\frac{-t}{R_{L}C}\right)\right]$$

**Figure 3:**
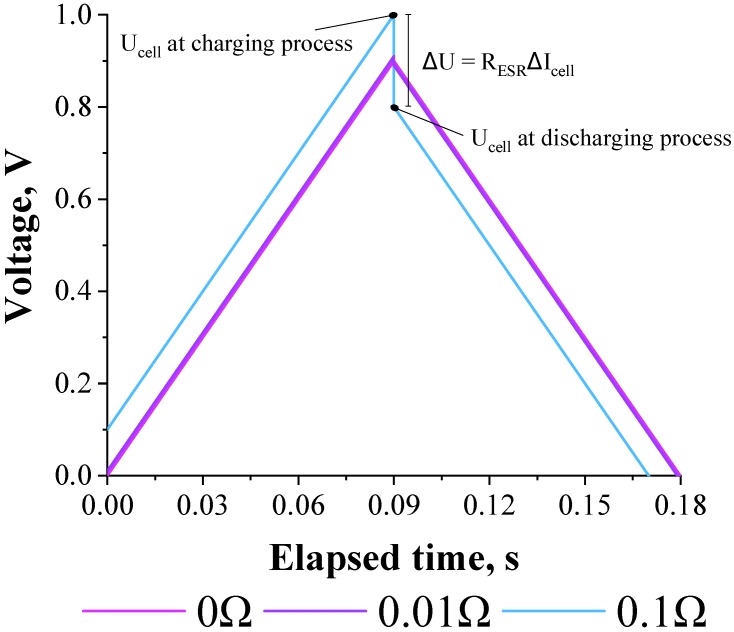
Simulation of the galvanostatic charge–discharge curves using different values of *R*_ESR_. Conditions: *R*_ESR_ = 0, 0.01, and 0.1 Ω; *R*_L_ = 1.0 MΩ; and *C*_EDL_ = 0.1 F. The anodic (positive) and cathodic (negative) currents were alternated between +1 A and −1 A, respectively.

Using Equations (1) and (2), the theoretical galvanostatic charge–discharge curves accounting for the charging and discharging processes on *C*_EDL_, respectively, were obtained at constant current from numerical simulation (e.g., Simulink of PSIM software) using different *R*_ESR_-values (e.g., 0, 0.01, and 0.1 Ω) keeping the *R*_L_ = 1.0 MΩ and *C*_EDL_ = 0.1 F (see Figure 3). The anodic (positive) and cathodic (negative) currents were alternated between +1 A and −1 A, respectively. In this case, a virtual controlled current source was applied referring to a charging time of ~1 s. Then, the current direction was reversed, thus characterizing the discharge of the *C*_EDL_. The magnitude of the charge and discharge currents was maintained so that the desired characteristics of the circuit could be graphically observed.

The *R*_ESR_-value can be determined by the voltage drop during the reversal of the polarity, i.e., when the charging process is discontinued to obtain the discharging curves. In the case of the cell voltage during the charging process (see Equation (1)), when *t* → ∞ the capacitor is fully charged. Therefore, one has for this particular condition that:

(3) $$U_{\mathrm{cell}(t)}=R_{ESR}I_{\mathrm{cell}}+R_{L}I_{\mathrm{cell}}$$

Remembering that for the charging voltage when *t* → ∞ one has that *R*_L_*I*_cell_ is equal to the voltage in the capacitor (*U*_SC_^0^), the following equation can be obtained:

(4) $$U_{\mathrm{cell}(t)}=R_{ESR}I_{\mathrm{cell}(\mathrm{charging})}+U_{\mathrm{sc}}^{0}$$

In the case of the cell voltage for the discharging process (*t* → 0), when *t* tends to zero the capacitor is fully discharged. Thus, for these conditions one has from Equation (2) that:

(5) $$U_{\mathrm{cell}(t)}=U_{\mathrm{sc}}^{0}-R_{ESR}I_{\mathrm{cell}(\mathrm{discharging})}$$

Calculating the difference between Equations (1) and (2), the following relation is obtained:

(6) $$\Delta U=R_{ESR}\left(I_{\mathrm{cell}(\mathrm{charging})}+I_{\mathrm{cell}(\mathrm{discharging})}\right)=2R_{ESR}\left|I_{\mathrm{cell}}\right|$$

Since the charging and discharging current are equal in modulus. Finally, the theoretical expression for the equivalent series resistance is given by Equation (7):

(7) $$R_{ESR}=\frac{\Delta U}{2\left|I_{\mathrm{cell}}\right|}$$

Therefore, it become obvious from Equation (7) that the voltage drop must be normalized by a factor of 2 in order to obtain reliable findings during application of the GCD method. In a Supplementary Data we performed an alternative approach for same demonstration, please have a look.

### 3.2. Validation of the Theoretical Expression Obtained for the Equivalent Series Resistance Using a Commercial Supercapacitor of 200 F

**Figure 4:**
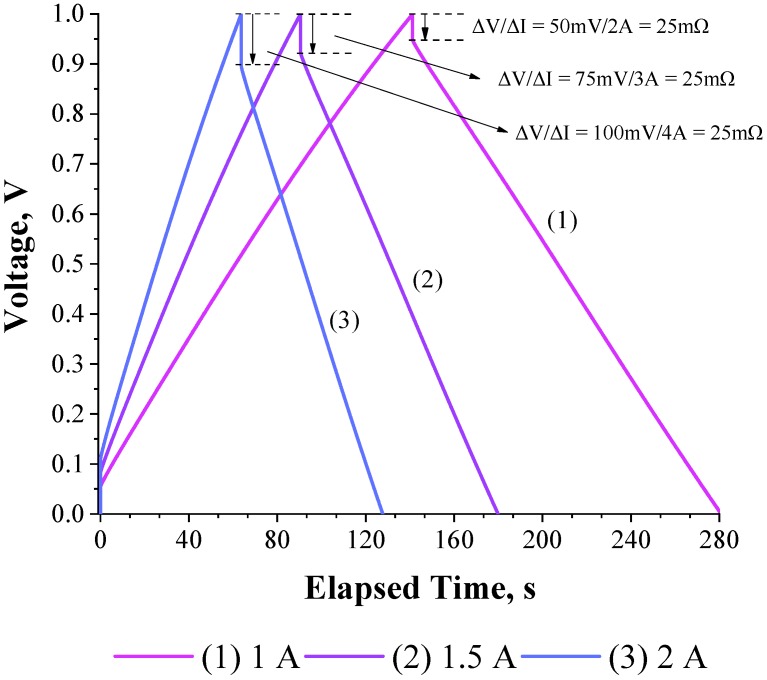
Experimental GCD curves obtained for a 200F commercial supercapacitor (2.7V D35H62 PTH S0016) as a function of the applied current.

Figure 4 shows that the GCD curves obtained for a 200 F commercial supercapacitor (e.g., 2.7V D35H62 PTH S0016) as a function of the applied current (e.g., 1–2 A). For these high values of the applied currents the Δ*U*/Δ*I* ratio was constant (=25 mΩ) [23].

**Figure 5:**
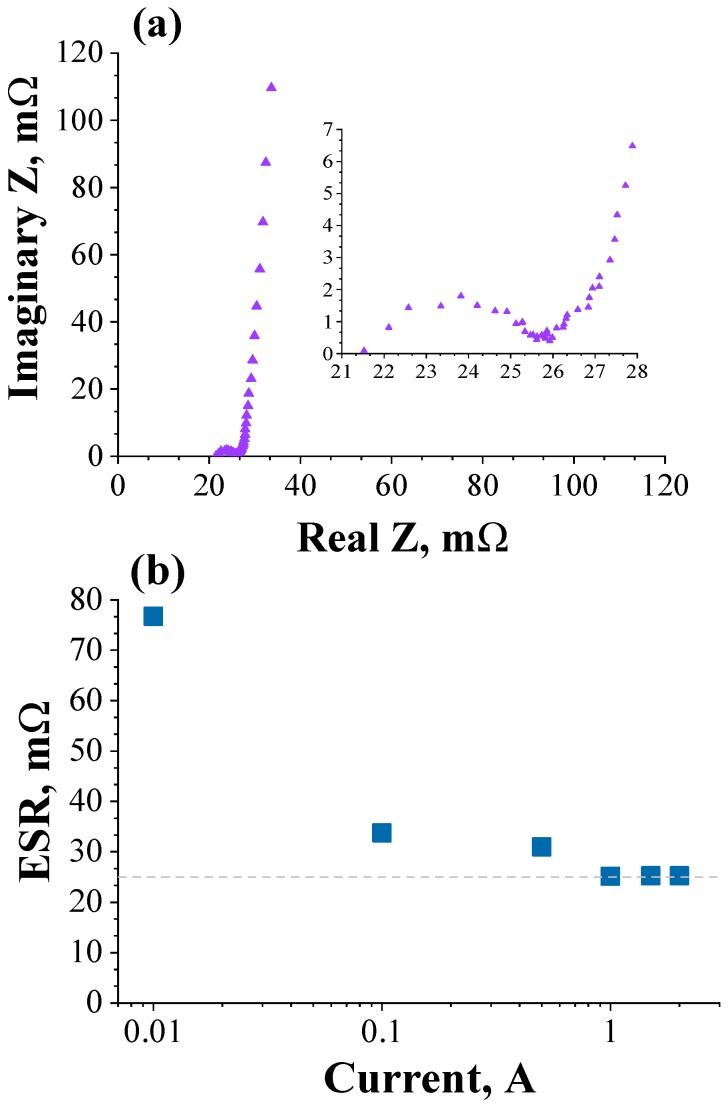
(**a**) Nyquist plot obtained for the commercial supercapacitor of 200 F. (**b**) Equivalent series resistance obtained from the GCD method as a function of the applied current.

Figure 5a shows the Nyquist plot obtained for the 200 F commercial supercapacitor (experimental conditions: δ*U* = 10 mV (peak-to-peak), *U*_d.c._ = 0 V, and Δ*f* = 10 kHz to 10 mHz). The impedance data of a real electric double-layer capacitor (EDLC) were quite different from the impedance response of a conventional (passive) capacitor due to the presence of the frequency dispersion phenomenon [4,17]. In fact, as discussed earlier by Conway [4], the EDLCs composed of high surface area porous carbon materials cannot be represented by a simple capacitance or even by a simple *RC* circuit due to the influence of the ions in conjunction with the porous behavior exhibited by the electrode material, i.e., the high-frequency voltage hardly penetrates inside the narrow pores while the low-frequency voltage penetrates inside the porous electrode structure.

Figure 5a shows that at high frequencies the Nyquist plot was characterized by a semicircle which indicates the existence of a Faradaic leakage resistance in parallel with a capacitive element as a consequence of the contributions from the pseudocapacitance associated with surface redox functionalities present at the interfaces and/or edges of carbon particles [5]. Obviously, in practice, the *R*_ESR_-value is obtained at very high frequencies from extrapolation on the real axis of the Nyquist plot. Thus, it was verified in the present case that *R*_ESR_ = 22 mΩ, which is in good agreement with that of 25 mΩ obtained in this work using the classical GCD method.

At moderate frequencies, there was a linear region in the Nyquist plot with a phase angle of approximately −45°, which indicates the presence of electrochemically active pores in the electrode structure. This characteristic behavior exhibited by porous electrodes was previously discussed in the literature [6]. On the contrary, the existence of a Warburg impedance (W), which is also characterized by a phase angle of approximately −45°, indicates that the electrochemical process is diffusion controlled during oxidation/reduction of the surface redox functionalities. In these different cases, the charge storage process is distributed over a network of *R* and *C* elements commonly represented by a semi-infinite transmission line of continuously connected *R* and *C* components. Despite the these considerations, one has in the special case of EDLCs characterized by a phase angle at high-frequencies of approximately −45° that is more plausible from the physicochemical viewpoint to consider the major influence of the porous electrode structure instead of a mass-transport controlled process since the real influence of the pseudocapacitance caused by the surface redox functionalities can be negligible in comparison with the purely electrostatic capacitance caused by the accumulation of the ions at the electrode/solution interface.

In addition, the analysis of the experimental findings obtained at low frequencies (see Figure 5a) revealed the presence of an inclined capacitive line. In fact, this behavior is predicted by the De Levie’s model representing the electrochemical response of porous electrodes [7]. Considering the presence of non-idealities due to the frequency dispersion phenomenon, the inclined capacitive line can be represented by a R*-*CPE* circuit model where *Z*_CPE_ = 1/*Y*_o_(*jω*)*^n^* and *n* ≅ 1. In this case, R* contains information about the electrolyte resistance inside the pores while the parameter *Y*_o_ represents the non-ideal capacitance.

Figure 5b shows the *R*_ESR_-values obtained from the GCD method as a function of the applied current. As it can be seen, a stationary value of 25 mΩ was obtained for applied currents higher than 1 A. These findings are in agreement with the experimental value of 22 mΩ obtained using the EIS technique. It must be emphasized that for an applied current lower than 1 A the *R*_ESR_ values were overestimated in comparison with those obtained with the EIS technique. Therefore, the present study suggests that reliable values of the *R*_ESR_ must be obtained in the case of the GCD method using different currents in order to verify a stationary (constant) value. To the best of our knowledge, this important issue regarding the influence of the applied current on the *R*_ESR_ values has not been addressed in the literature.

## 4. Conclusions

From the theoretical analysis of a canonic circuit model an Ohm-like equation was deduced for the determination of the equivalent series resistance (*R*_ESR_) using the galvanostatic charge–discharge method. It was verified that the voltage drop must be normalized by a factor of two in order to obtain meaningful findings. The present work compared the applications of the electrochemical impedance spectroscopy (EIS) technique and galvanostatic charge/discharge (GCD) method in order to determine the value of the equivalent series resistance (*R*_ESR_) present in electric double-layer capacitors (EDLCs). The derived equation was applied to obtain the *R*_ESR_ of a 200 F commercial supercapacitor.

In principle, the theoretical treatment presented in this work is strictly valid for EDLCs, i.e., where the battery-like Faradaic reactions are absent. Therefore, we may find discrepancies during the analysis of the experimental findings obtained for pseudocapacitors (PCs) where solid-state surface redox reactions occur. Further complications can also appear in the case of asymmetric capacitors since in these cases the positive and negative electrodes are not identical (i.e., they are composed of dissimilar materials).

## Supplementary Materials

The following are available online.
