# Supplementary material for: How to Measure and Calculate Equivalent Series Resistance of Electric Double-Layer Capacitors
Source: Molecules. 2019 Apr 12;24(8):1452. doi: 10.3390/molecules24081452 (PMC6515551; doi:10.3390/molecules24081452)
Supplement: Supplementary file 1 [file molecules-24-01452-s001.pdf]

Supplementary information

Alternative theoretical approach for deriving an expression for the R_{ESR}

There is another way to determine voltage drop (ΔU), which is based on the continuity of the voltage in a capacitor. Figure 1Sa shows the equivalent circuit now connected to a current source while the plot in Figure 1Sb shows the profile of the square current wave used in the present case.

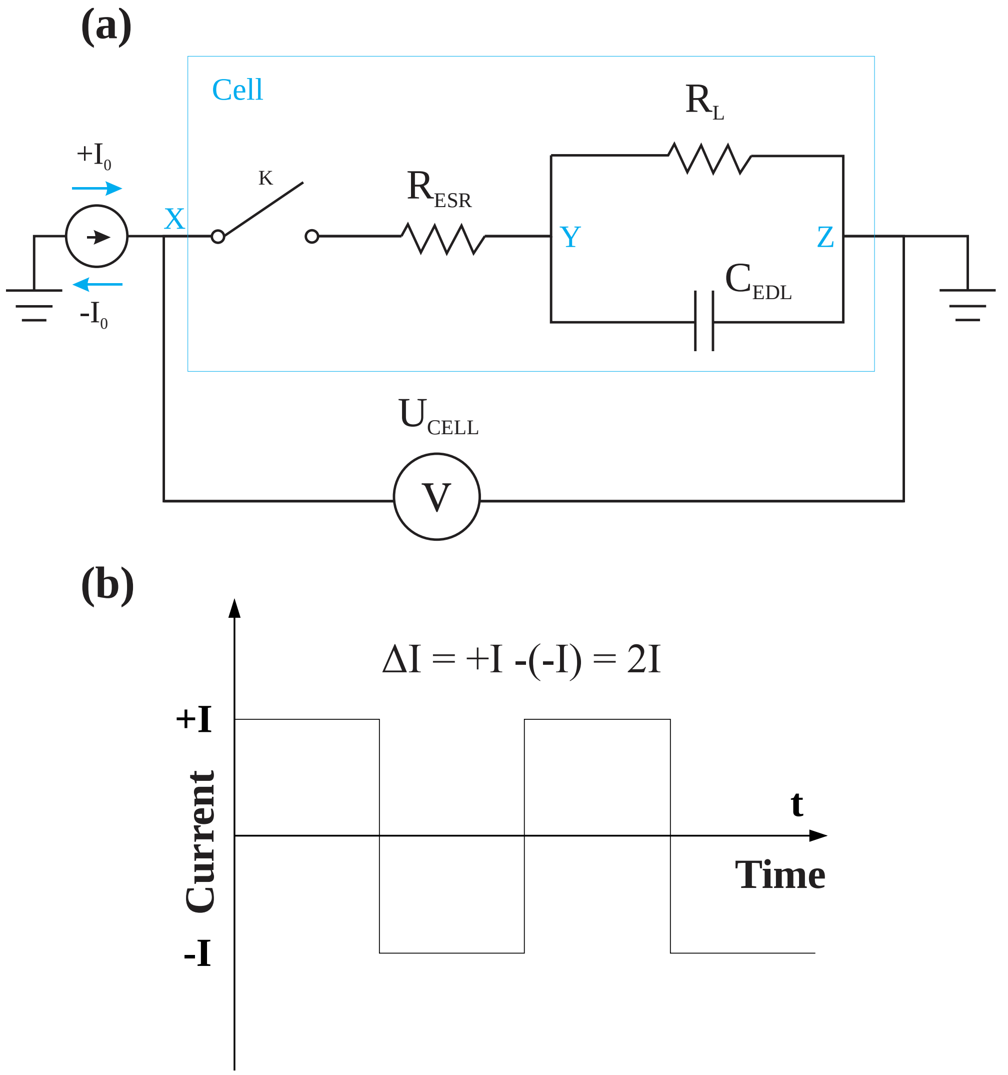

Figure 1. The equivalent circuit connected to a current source (**a**) and the plot (**b**) showing the profile of the square current wave.

Notice that times t_0, t_1, and t_2 are the switching times. When the current is switched at an instant t, the voltage on the capacitor C cannot change abruptly. Therefore, one has

that $U_{C(t-)} = U_{C(t+)}$. With this idea in mind, it is easy to derive an expression for Δ*U*. Before t_0, the current is zero and the capacitor is initially discharged $U_C = 0$.

In addition, the elements *C* and R_L are in parallel, thus U_c = U_{RL} = U_{yz} and $U_{xy} = U_{ESR} = 0$. In this way the total cell voltage for just before t_0 is

$$U_{cell(t_0-)} = U_{xy} + U_{yz} = 0 \tag{1}$$

At t_0, a current $+I_0$ starts to flow from the left to right. U_{xy} becomes instantaneously equal to $+I0R_{ESR}$, but U_c remains zero since it cannot change abruptly. Therefore, one has that:

$$U_{C(t-)} = U_{C(t+)} = 0 = U_{yz} \tag{2}$$

At t_0^+ the total voltage of the cell goes up to:

$$U_{cell(t_0+)} = U_{xy} + U_{yz} = I_0 R_{ESR} + 0 = I_0 R_{ESR} \tag{3}$$

Therefore, one has that the *first change* in cell voltage due to the application of the positive current is given by:

It is worth mentioning that this voltage drop occurs only during the experiment (e.g., for an interval of *T*/2 of the square wave, where *T* is the period) while the current goes from zero up to $+I_0$. Therefore, the next voltage drop must be twice this value as will be shown in this section. Probably, this important fact is the source of the error present in many scientific papers.

Then capacitor *C* will be charging. There will be a transient period when U_c will increase and a stationary period when the U_c will reach a constant value. At the instant t_1, the current will switch again its polarity and, therefore, U_{xy} becomes instantaneously equal to $-I_0R_{ESR}$. Either for the transient or stationary periods, U_c will be $U_c(t_1^-)$ before switching the current polarity, i.e., U_{RL} will also be U_c since they are in parallel.

Considering that U_c will not change immediately during the switching process, one must have the condition that $U_{C(t-)} = U_{C(t+)} = K$. As a result, the following relation can be obtained:

$$U_{cell(t_1+)} = U_{xy} + U_{yz} = -I_0 R_{ESR} + U_{C(t_1-)} = -I_0 R_{ESR} + K \tag{4}$$

Finally, the voltage drop due to R_{ESR} can be given as follows:

$$\Delta U = U_{cell(t_1-)} - U_{cell(t_1+)} = I_0 R_{ESR} + K - (-I_0 R_{ESR} + K) \tag{5}$$

$$R_{ESR} = \frac{\Delta U}{2 I_0} \tag{6}$$

Conversely, after the switching, capacitor C will discharge and charge in the opposite directions. At t_2, the current switches again and U_{xy} becomes instantaneously equal to $+I_0R_{\mathrm{ESR}}$. Regardless the electric system is in the stationary or transient regime, U_{c} will have a certain value immediately before instant t_2 and this value will have to be the same just after t_2:

$$U_{C(t_2-)} = U_{C(t_2+)} = L \tag{7}$$

Immediately before t_2, one obtains the following relationship:

$$U_{cell(t_2-)} = U_{xy} + U_{yz} = -I_0 R_{ESR} + U_{C(t_2-)} = -I_0 R_{ESR} + L \tag{8}$$

Immediately after t_2, one obtains the next relation:

$$U_{cell(t_2+)} = U_{xy} + U_{yz} = +I_0 R_{ESR} + U_{C(t_2+)} = +I_0 R_{ESR} + L \tag{9}$$

Again, the voltage drop due to R_{ESR} can be given as follows:

$$\Delta U = U_{cell(t_2+)} - U_{cell(t_2-)} = I_0 R_{ESR} + L - (-I_0 R_{ESR} + L) \tag{10}$$

$$R_{ESR} = \frac{\Delta U}{2 I_0} \tag{11}$$

The same physical situation will repeat for the next switching times during the application of the square current wave.
